# On Rest and Pain

**Published:** 1879-04

**Authors:** 


*On Rest and Pain.—A Course of Lectures on the Influence of Mechanical and Physical Rest in the Treatment of Accidents and Surgical Diseases, and the Diagnostic Value of Pain.* By JOHN HILTON, F. R. S., F. R. C. S., Surgeon Extraordinary to Her Majesty the Queen. New York: William Wood & Company, 27 Great Jones Street.

This is the first volume of Wood's Library of Standard Medical Authors, which has been offered to the profession at such prices as to attract the attention of the profession everywhere. Twelve volumes to complete the Library for the astonishing low price of twelve dollars for the entire number. This first volume speaks favorably for the enterprise. It is practically valuable, and its subject matter should be studied by all physicians.

